# GnRH-immunocastration: an alternative method for male animal surgical castration

**DOI:** 10.3389/fvets.2023.1248879

**Published:** 2023-10-31

**Authors:** Chun Wang, Cuiting Yang, Yutian Zeng, Ming Zhang

**Affiliations:** ^1^College of Animal Science and Technology, Sichuan Agricultural University, Chengdu, China; ^2^Key Laboratory of Livestock and Poultry Multi-Omics, Ministry of Agriculture and Rural Affairs, College of Animal Science and Technology, Sichuan Agricultural University, Chengdu, China; ^3^Farm Animal Genetic Resources Exploration and Innovation Key Laboratory of Sichuan Province, College of Animal Science and Technology, Sichuan Agricultural University, Chengdu, China

**Keywords:** immunocastration, surgical castration, male animal, animal welfare, GnRH

## Abstract

Castration of male animals is intended to produce high-enhance quality of animal meat, prevent unpleasant taste, reduce aggressive behavior, and manage overbreeding. Over the years, Tranditional methods of mechanical and surgical castration have been employed over the years, but they fall short of meeting animal welfare requirements due to the associated risk of infection, pain, and stress. Immunocastration, specifically Gonadotropin-releasing hormone (GnRH)-immunocastration, targeting the hypothalamic–pituitary-testis (HPT) axis, has emerged as an animal-friendly alternative to surgical castration, effectively addressing these issues. This review seeks to systematically summarize the principles, development, current applications and challenges of GnRH-immunocastration, offering insights into its role in promoting animal welfare.

## 1. Introduction

Capon production, an ancient practice dating back over 3,000 years, persists globally (1–3). While capon production constitutes a modest segment of the market, it holds significant growth potential due to its dustinctive sensory attributes cherished by consumers (4–7). Capon are male chickens that undergo surgicalcastration before reaching sexual maturity, a practice also applied to other male livestock like boars and rams. The objectives are to reduce unpleasant odors, increase intramuscular fat deposition, improve carcass composition and meat quality. Castration leads to androgen deficiency, hindering male secondary characteristics, such as the comb and flesh hair, reducing aggressive behaviors and eliminating fighting and snorting (8). The energy consumed by capon in territorial protection, fighting, and courtship behaviors is greatly reduced compared intact rooster, making their feed energy utilization more efficient for growth and fat deposition (9). Consequently, castration enhances fat deposits and intramuscular fat content, elevating meat sensory qualities such as tenderness, juiciness, and flavor (7, 9–11). However, surgical castration also has some limitations, including postoperative complications, increased susceptibility to infections, and animal welfare concerns. Additionally, the procedure needs to be performed at an appropriate age, and the high demands on surgical skills, and other cost-effective resources (12). In contrast, GnRH-immunocastration minimizes animal stress, reduces infection risk and complications associated with surgery, and substantially greatly improves animal welfare. Furthermore, it poses no risk of drug residue, making it easy to apply in production. Consequently, GnRH-immunocastration has the potential to be a safe alternative to surgical castration.

## 2. The comparison of different castration techniques for male animals

Currently, various techniques for castrating male animals exist, including chemical castration, Bloodless castration, surgical castration, and immunocastration (Table 1). Unlike mammals, rooster’s testicles are located in the abdominal cavity, hanging ventral in the anterior part of the kidney through the mesangium and with the posterior tibial vein and aorta on both sides, which makes avian castration is more challenging than that of mammals. In the poultry industry, traditional surgical castration is performed without anesthesia or analgesic control, resulting in roosters’ suffering and violating animal welfare principles (38). Although geldings are banned in the EU (European Union) due to concerns about animal welfare, they are still used in traditional agricultural systems, representing a derogatory toward age-old practices (39). Surgical castration also incurs mortality rates ranging from 5 to 20%, and sometime even up to 50% (39).

**Table 1:** Comparison of four castration techniques.

Item	Method			
	Surgical castration	Bloodless castration	Chemical castration	Immunocastration
Principle	Cutting, and removing the gonads	Rubber ring, pliers or Burdizzo castration, blocking the scrotum or spermatic cord, preventing blood flow and causing necrosis	Injection of chemical drugs(Lactic acid, phenol, benzyl alcohol, etc.), causing testicular parenchymal lesions, inhibiting testicular function	Using exogenous reproductive hormone targets to produce antibodies *in vivo*, combined with endogenous hormones, lowering testosterone levels and suppressing testicular development and spermatogenesis
Characteristics	It demands someone skilled in castration techniques to operate	Simple and economical to operate; suitable for young animals; irreversible permanent castration	Low stress, no drug residue, inexpensive, less time consuming, easy to perform	Suitable for both young and late production; strengthen immunity according to demand
Castrated animals	Pigs, Chickens, Cattle, Sheep, Foals	Cattle，Sheep	Dog, Cat	Pigs, Chickens, Sheep, Cattle, Mice, Horses, Deer, Cat
castration effect	Irreversible, The castration is clean and thorough	Irreversible, The castration is clean and thorough	Irreversible; Insufficient injection or inaccurate injection site can lead to castration failure	Reversible, The castration effect is mild, and a small number of animal individuals fail to respond effectively to antigens, resulting in immune failure
Anesthesia	Yes	No	No	No
Reaction	Increase animal stress, produce pain, postoperative infection and cause complications	Strong pain, long duration, stress response	No pain	No pain
Side effect	Prone to postoperative bleeding, causing infection and complications	Improper operation leads to cessation of blood supply to the bottom of the scrotum and secondary tissue necrosis	Inaccurate injection sites can lead to degeneration of other tissues	Slight redness and inflammation at the injection site
Animal welfare	No	No	Yes	Yes
Cost	Lower	Lower	High	High
Reference	(7, 13–20)	(21–24)	(25, 26)	(27–37)

## 3. Principles of GnRH-immunocastration

**Figure 1:**
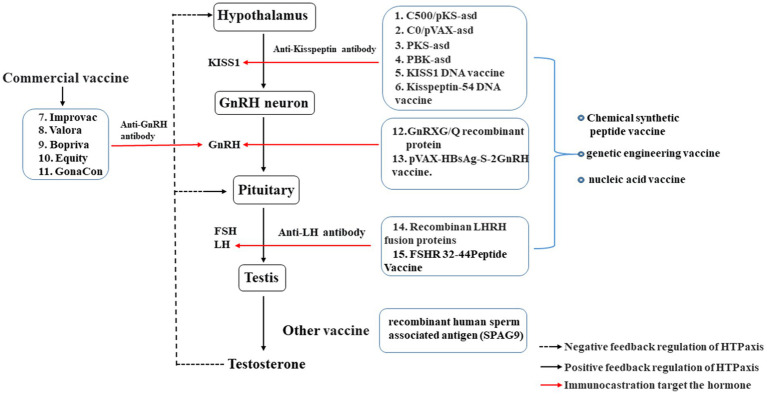
Effect of immunocastration on the hypothalamic–pituitary-testicular axis. The vaccines: 1–6, Immunocastration vaccine targeting Kisspeptin; 7–13, Immunocastration vaccine targeting GnRH; 14–15, Immunocastration vaccine targeting LH.

Immunocastration primarily targets reproductive hormones within the HPT axis (Figure 1), disrupting reproductive hormone within the HPT axis through immunological means to reduce the concentration of target hormones and achieve castration (29, 40). GnRH is located at the upper end of the HPT axis, plays a pivotal roleinitiating and controlling the physiological functions of the entire reproductive axis (41). Therefore, GnRH-immunization is the most widely used in production compared other targeted hormone immunocastration involves Animals are inoculated with GnRH vaccine, which prompts the production of specific anti-GnRH antibodies in the body, anda lot of anti-GnRH antibodies bind with endogenous GnRH, continuously inactivating endogenous GnRH. Consequently, GnRH-immunization leads to a decrease in luteinizing hormone (LH) and follicle stimulating hormone (FSH) secretion. Eventually, this inhibition of animal gonadal functionresults in the achievement of castration (42).

## 4. GnRH-immunocastration is a safe castration method in line with animal welfare

Physiological doses of GnRH can significantly increases LH levels and slight increase FSH levels in plasma, reaching the gonads via the pituitary portal circulation, This stimulated the synthesis and secretion of gonadal steroid hormones, promoting gonadal development, gamete production, and the occurrence and maintenance of secondary sexual characteristics. GnRH immunocastration induces a lot of GnRH-antibodies that neutralize endogenous GnRH, and the production of the antibody is a sustained biological effect. As a result, GnRH immunocastration consistently inhibit testicular or ovarian endocrine function, reducing hormone levels and reproductive activity, and associated odors, primarily skatole and androstenone (43–46). Immunization with GnRH leads to a substantial decrease in androstenedione and testosterone in male animals (29, 32, 34, 47, 48). Consequently, European countries are advocating for GnRH-immunocastration as a surgical castration alternative, improving animal welfare. Immunocastration alleviates animal stress, reduces the risk of infection and complications associated with surgical castration, reduces pain and enhances animal welfare. GnRH-immunocastration is considered relatively safe alternative to surgical castration.

## 5. Current application of GnRH-immunocastration vaccine

Immunocastration is not limited to pork production but is also employed in other livestock animals as an alternative to surgical castration. Its key advantage lies in eliminating pain, wound infection risks, and potential losses associated with castration (49). Now, several commercial immunocastration products have been applied in animal production (Table 2). However, in Europe, Improvac is the sole product approved for commercial use in pigs., yet its market share is only 2.8% of all male pigs, despite EU approval almost a decade ago. Belgium produces about 15% of the castration vaccine in Europe, while globally, Brazil and Australia hold a market share of more than 50% (55, 56).

**Table 2:** Several commercial immunocastration products on the market.

Vaccine	Supplier	Indication/Target species	Formulation	Doses	Reference
Improvac/Valora	Ceva Santé Animale, Libourne, France	Immunocastration and prevention boar taint/swine	Active substance: Gonadotropin releasing factor (GnRF) analog-protein conjugate +Adjuvant: 300 mg Diethylaminoethyl (DEAE)-Dextran +2 mg Chlorocresol	Two subcutaneous doses, at least 4 weeks apart.	(50)
Bopriva	Zoetis, Parsippany, NJ, United States	Immunocastration, fertility control/cattle	400 μg GnRH–protein conjugate	Two doses at an interval of 3 weeks with 1 mL Bopriva	(51)
Equity	Zoetis Inc., Parsipanny, New Jersey, United States	Control of estrus in horses and deer	300 μg Iscomatrix (dipalmitoylphosphatidyl choline + Saponin Quil A + Cholesterol) + 200 μg GnRH–DT	Two doses, at least 4 weeks apart.	(52)
GonaCon	USDA, Pacarello, ID, United States	Fertility control/white-tailed deer, wild boar, horses, feral cattle, and bison, prairie and feral dogs, feral cat both sexes	GnRH–protein conjugate concentration:1,000 μg/mL Concholepas concholepas haemocyanin +AdjuVac™ (mineral oil-based) +166 μg/mL *Mycobacterium avium* concentration	Single dose	(53, 54)

## 6. Current challenges for GnRH-immunocastration in male animals

**Figure 2:**
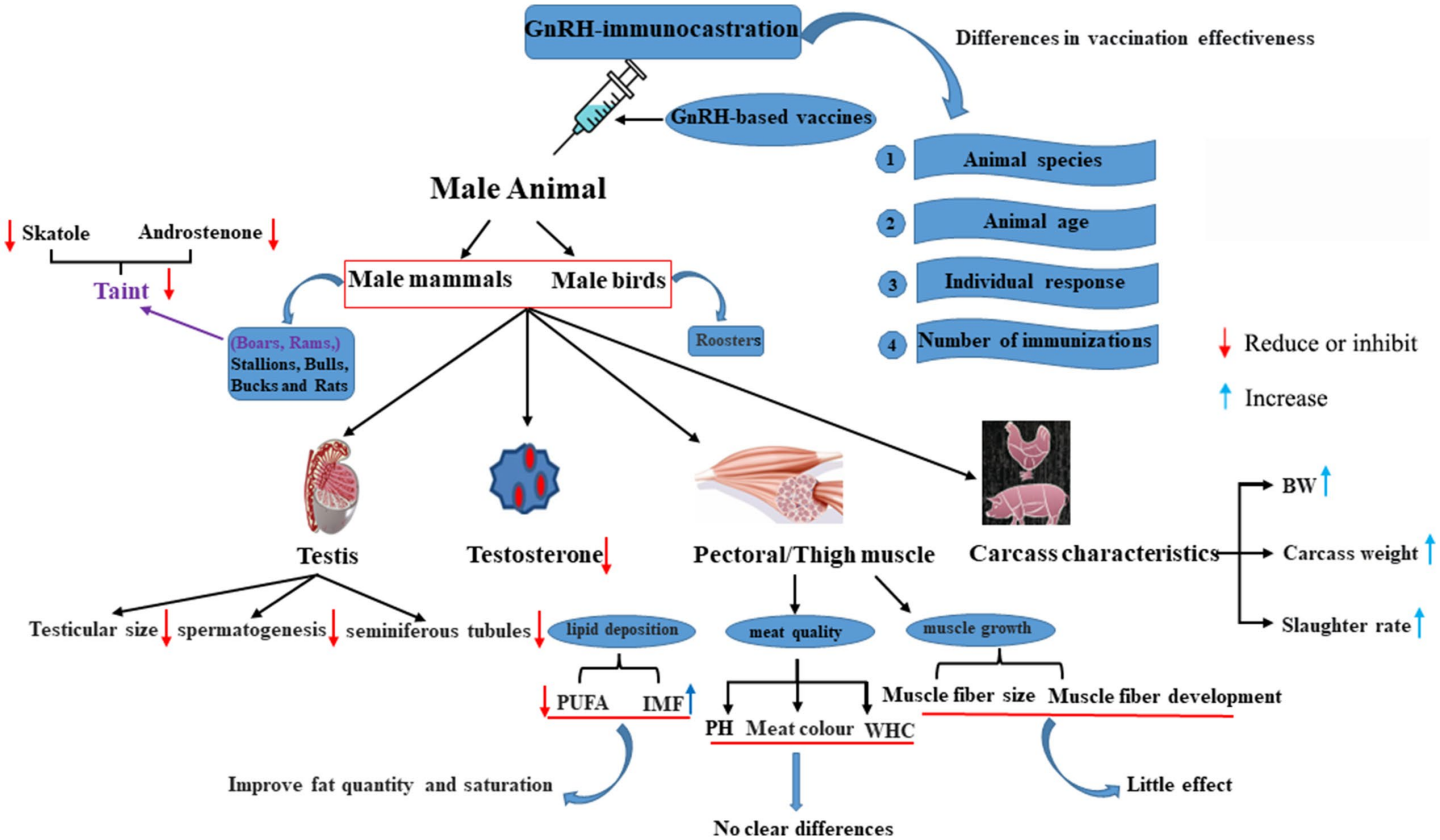
GnRH-immunocastration effects across diverse animals. PUFA, polyunsaturated fatty acids; WHC, water holding capacity; BW, body weight; PH, Pondus Hydrogenii.

Immunocastration, often administered using the GnRH vaccine, has undergone extensive investigation in male mammals and birds (Figure 2) (43, 57–61). Outcomes vary based on the animal species, animal age, individual response, and immunization frequency (62). GnRH plays a crucial role in regulating gonadal development and function through the pituitary gland. GnRH-immunocastration significantly decreases reproductive performance of male animals by inhibiting the development testes. Studies have confirmed that immunizing male animals with GnRH can cause infertility, gonadal atrophy, and changes in meat quality by directly or indirectly acting of testosterone (63).

### 6.1. In male mammals

In mammals, the majority of research on GnRH-immunocastration has focused on male animals, and spermatogenesis is inhibited after GnRH immunization. For example, immunizing male animals such as boars, bulls, stallions, rams, bucks, and rats with GnRH vaccine leads to the suppression of testicular, epididymal, and vas deferens development. This results in reduced sperm concentration in the testicles with low viability, constriction of the seminiferous tubules, and inhibition of spermatogonia and spermatoblast production in the deep epithelium (29, 59, 60, 64–68).

At present, GnRH immunocastration is the most widely used in boars. Androstenone is a male hormone that is formed in the cells of the Leydig and has a urine-like odor (69). Skatole is a metabolite of the amino acid tryptophan with a fecal odor that is synthesized by microbial degradation in the colon (44, 70). Immunocastration has been shown to effectively prevent the accumulation of boar taint in adipose tissue by reducing steroid hormone synthesis in the testes (45). However, due to the short duration of the castration effect, the control of boars taint requires multiple doses of GnRH vaccine, and the second vaccination is often carried out 4–6 weeks before slaughter in production, and even the third dose of vaccine is required for slaughter pigs with higher age and weight to control boars odor, which increases the cost.

Meat quality is increasingly valued by consumers, so male livestock are castrated in production to improve meat quality. Currently, a large number of studies have focused on the improvement of meat quality through immunocastration. GnRH immunocastration reduces the accumulation of taint compounds in adipose tissue and improves meat quality and carcass characteristics in male mammals (71). However, the latest study found that the slaughter rate of immunocastrated boars is lower than that of surgical castrated boars and intact boars, as immunocastrated boars have heavier liver and kidneys (72). The abdomen of immune castrated pigs is fatter than that of entire boars, and the lean meat rate is similar to that of surgical castrated pigs, both of which are lower than that of entire boars. Therefore, to some extent, it will affect consumers’ choices. Bellies from immunocastrated pigs are fatter and firmer than those from boars. In addition, although immunocastration increases intramuscular fat content and reduces polyunsaturated fatty acids, the effect of improving intramuscular fat is still not as effective as surgical castration, and boars that undergo surgical castration have lower polyunsaturated fatty acids (73). Similarly, studies have shown that compared to surgical castration, GnRH immunocastration improves cattle weight, but there are no differences in beef pH, color, fat coverage, cooking loss, or tenderness (74).

### 6.2. In male birds

The utilization and assessment of immunocastration vaccines in pigs has been extensively reported (34, 48, 75). However, there is currently no commercially available vaccine for chickens. Recently, only three studies have investigated the use of the GnRH vaccine for immunizing roosters. Quaresma and colleagues evaluated the effects of Improvac on the body and bone development, meat color, and composition of roosters, and found that the color parameters of Improvac birds, such as brightness, red, and hue angle, were between roosters and capons (5). In addition, i.c. Antunes et al. found that immunocastration had little effect on the fatty acid profile of broilers, but improved overall lipid markers in breast and leg meat to some extent, which could partially enable GnRH immunization (6). Previous studies have shown that both caponization and ovariectomy likely improve the meat quality of the breast muscle based on the objective indices of IMF, appearance (color), texture, and minor change of the fatty acid profile; ovariectomy improves flavor-related indices (76). In our study, we found that roosters inoculated with Improvac had some effect on muscle development, but the effect was not completely satisfactory (77, 78).

## 7. Conclusions and perspective

Immunocastration currently faces challenges related to immunization failure. These challenges include significant variations in individual responses among immunized animals, insensitivity to antigens in some individuals, failure to elicit an immune response, or a shorter duration of immune effect. This shorter duration leads to an increase in testosterone concentration during the recovery period compared to the previous phase, resulting in a gradual return of sexual behavior. Multiple vaccinations are necessary to counter this effect, which in turn escalates costs. Moreover, there are associated disadvantages for farmers, including increased expenses for purchasing produce and labor management, the risk of accidental self-injection by farm workers, and uncertainty regarding consumer attitudes toward meat from pharmacologically castrated animals. However, it’s important to note that immunocastration offers several advantages, such as reducing animal stress, lowering the risk of infections and complications associated with surgical castration, significantly improving animal welfare, and being relatively straightforward to implement in production settings. Therefore, immunocastration may remain a safe alternative to surgical castration in the future.

In the future development of commercial castration vaccines, particularly GnRH immunocastration vaccines for male animals, there should be an exploration of the construction of immunogens, immune dosages, immune strategies, and timing. Attention should be directed toward enhancing the effectiveness and prolonging the duration of immune response for these vaccines. Currently, research on GnRH vaccines primarily focuses on chemical synthesis of polypeptides, dual conjugate vaccines, DNA vaccines, tandem conjugate vaccines, among others. However, these approaches have their limitations. Considering the existing challenges with GnRH gene vaccines, it’s worth considering research and development of GnRH gene engineering vaccines and GnRH recombinant adenovirus vaccines in the future. In summary, the future focus of immunocastration vaccine development will revolve around creating products with sustained immunogenicity, easy production, and stable effects. These advancements could hold the key to the future of immunocastration vaccines.

## Author contributions

CW: drafting the manuscript. YZ and CY: provision of study materials. MZ: conceptualization and supervision. All authors contributed to the article and approved the submitted version.
